# Catastrophic flooding effects on a Wisconsin wet prairie remnant: A shift in the disturbance regime?

**DOI:** 10.1371/journal.pone.0294359

**Published:** 2023-11-22

**Authors:** Paul H. Zedler, Bradley M. Herrick

**Affiliations:** 1 Nelson Institute, University of Wisconsin–Madison, Madison, Wisconsin, United States of America; 2 University of Wisconsin–Madison Arboretum, Madison, Wisconsin, United States of America; UNAM, MEXICO

## Abstract

Climate change is likely to imperil native biodiversity through the increased frequency of extreme events. Here we address the short-term effects of an extreme flooding event on an unplowed prairie reserve, the Faville Prairie Wisconsin State Natural Area. This 25-ha property is a remnant of the formerly extensive Crawfish Prairie that lay on the east bank of the Crawfish River, Jefferson County, Wisconsin USA. The Faville remnant has historically been subject to late winter to spring flooding in its lower portions. In June of 2008, however, an extreme rainfall event caused flooding unprecedented in the 87-year history of streamflow, inundating the entire site. Data were available from 180 permanently marked plots sampled in 1978–79. We assessed the change by resampling these plots in 2010–2015. At the m^2^ scale, we found significant losses of species richness, a result of most species having fewer occurrences than in the earlier data. There was near extinction of several important prairie species and a relative increase in wetland tolerant species. Lower elevation plots, subject to the encroachment of woody plants and the invasion of *Phalaris arundinacea* for decades prior to the flood, had the lowest levels of species richness. However, some prairie species survived the flooding with little change, and recent anecdotal observations show that others are rebuilding their populations. Thus, if extreme floods are infrequent, the prairie should be able to recover to its former state. If, however, the hydrological regime shifts toward more frequent, growing-season floods, we predict further decline in those plant species that were the object of the preservation of this remnant. It is critical that fire management continue along with monitoring to track species’ recovery or replacement, so that corrective measures can be identified and tested to sustain the native prairie species diversity.

## Introduction

Remnant communities, the bits of minimally disturbed natural ecosystems in humanized landscapes, play an important role in the struggle to ensure the survival of native biodiversity. It is increasingly recognized that grasslands, natural and restored, harbor a large proportion of the Earth’s biodiversity [1]. In the upper Midwest of the United States, this explains the concern for native grassland preservation. Recent research on prairie remnants in this region has, however, identified troubling trends. In many remnants there has been a gradual loss of species richness [2–4] attributed to the exclusion of fire and landscape fragmentation. The lack of fire is thought to limit the opportunities for recruitment of species [5–8]. Fragmentation and isolation both decrease the likelihood of fire and the possibility of reinvasion of lost species [9, 10]. They will also change the abundance and types of animal dispersal vectors [11]. An additional threat, which is relevant to the case to be discussed here, is the encroachment of the site by invasive nonnatives and native woody species [12, 13].

The Faville Prairie State Natural Area (hereafter: Faville Prairie) is an isolated unplowed remnant. However, contrary to the expectation of a gradual decline in biodiversity over time due to the remnant’s isolation, a resurvey in 2005 of permanent plots in the most species rich western portion of the site previously sampled in 1978–79 showed that the species richness of the site had changed little in that interval [14]. A reason for this may be that unlike many remnants, the Faville Prairie has benefitted from the reintroduction of fire, in part by accident when ditch burning by local farmers escaped onto the reserve land, and in part by purposeful use of prescribed burns by the managers of the site, the University of Wisconsin–Madison Arboretum [14]. Despite the retention of a significant level of biodiversity, however, the fires have not been sufficient to limit the increase of woody species or stop the intrusion of invasive non-native species, especially in the wetter portions of the site that were excluded in the 2005 resurvey.

This study was prompted by the occurrence of another potentially disruptive factor–extreme flooding that occurred in June of 2008 [15]. This flood was unique in the 74-year hydrological record of the Crawfish River for two reasons: 1) It was the most extreme, and 2) it occurred well into the growing season, rather than in late winter to early spring, as is more usual. Preliminary sampling of the site after the flood waters receded showed that this anomalous flood had had substantial effects on the vegetation.

Although it is difficult to ascribe any single event directly to climate change, there is an emerging consensus that flooding regimes are already showing the effects of climate change [16–20]. It has also been shown that the expected shift in flooding regimes towards larger floods reaching higher levels can have deleterious effects on the biota [21, 22]

The main questions motivating this research are descriptive: 1) To what degree were the different plant species in this prairie remnant affected by the flooding? 2) To what degree was the conservation value of the site changed? 2) Which factors might explain the differential survival of species? 4) A question that remains and is beyond the scope of this study concerns the capacity of the vegetation to rebuild after the flood induced changes. Will the depleted elements of the native prairie flora recover their former abundance and so preserve the biodiversity of this remnant?

## Methods

### Study area

**Fig 1:**
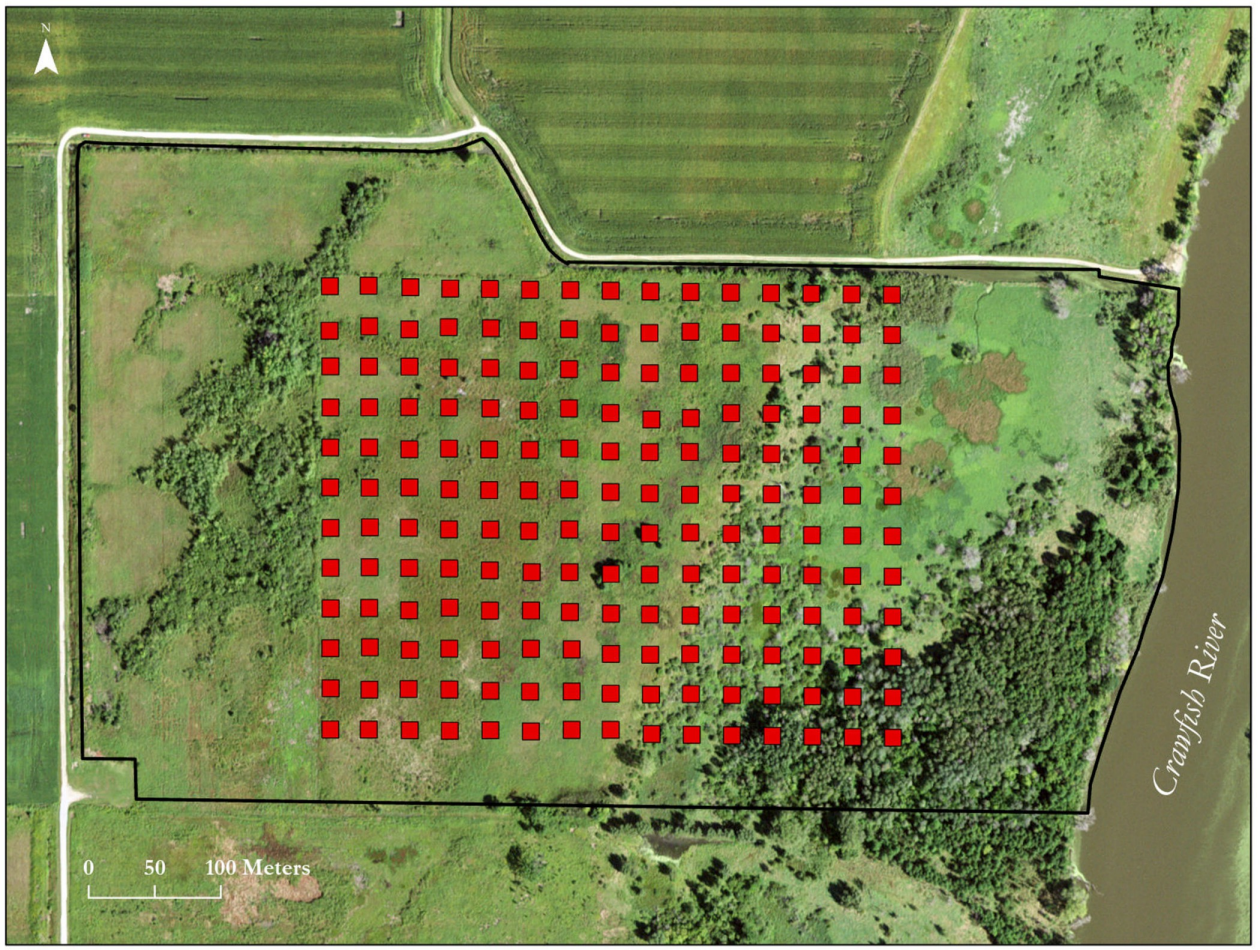
Aerial view of the Faville Prairie showing its location next to the Crawfish River. The black line indicates the borders. The arrow in the upper left points to North. The squares show the positions of the 180 permanently marked points established by Max Partch in 1947–48 which were the center of 4 m^2^ plots. These points were resampled in 1978–79, partially in 2005 [14], and again for the entire set in this study. The parcel bordering the Faville Prairie on the south is undergoing restoration by the Madison Audubon Society.

The Faville Prairie (43° 08’ 53” N, 88° 52’ 35” W) is a 35.4 ha property managed by the University of Wisconsin—Madison Arboretum. It is a roughly rectangular parcel oriented N-S and E-W, with its eastern boundary defined by the Crawfish River and its western and northern boundaries by gravel roads, farm fields, and associated drainage ditches (Fig 1). A 21-year ongoing prairie restoration managed by the Madison Audubon Society lies along its southern boundary.

There are two subdivisions of the Faville Prairie: an unplowed 25 ha remnant that was acquired in 1945, and 10.4 ha of former farmland added to the Reserve in the 1960’s. The partially restored vegetation of this later addition is not considered in this report.

The Faville Prairie is classified as wet to wet mesic prairie [23] grading to a wetland riparian zone along the Crawfish River. The average elevation from the western edge of the remnant to the riverbank drops by about 2 m. A ridge approximately 0.5 to 0.6 m above the adjacent land, presumably a legacy of post-glacial flooding, runs across the site and supports the greatest diversity of prairie species of most conservation interest (Fig 1). Portions of the site from the western boundary to the ridge have a “pitted” microtopography with many small depressions of unknown origin [24]. Following spring thaws and for prolonged periods in wetter years these depressions hold standing water.

The Faville Prairie is the largest remaining unplowed portion of the 1000-ha Crawfish Prairie which formerly lay on the west side of the Crawfish River. The historical lack of tree cover is attributed to recurrent fire, which prior to Euro-American settlement, would have usually spread from west to east under prevailing winds. Historically, and currently, the Faville Prairie is subject to flooding usually in late winter into early spring in its most riverward portions. At present the reach of the river that runs past the Faville Prairie is classified by the Wisconsin Department of Natural Resources as “impaired” due to high phosphorus levels and “significant polluted runoff impacts” including a high sediment load [25].

**Fig 2:**
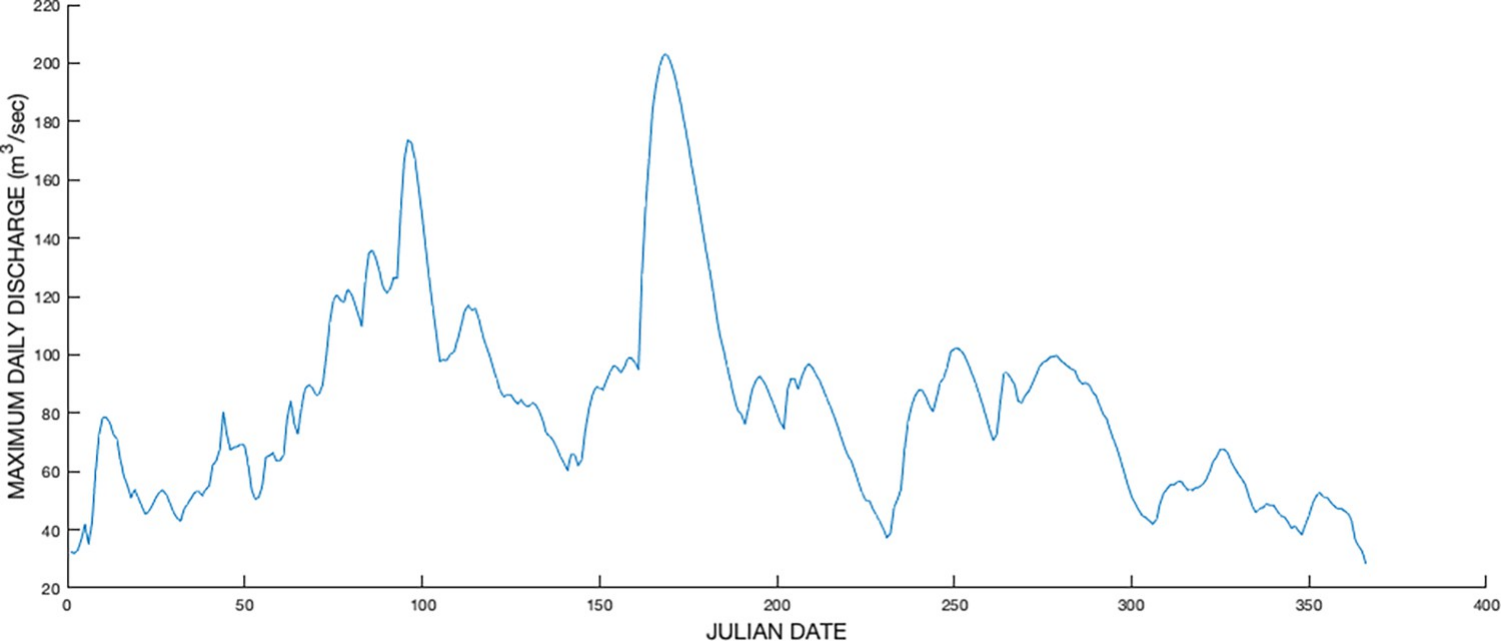
Daily maximum average discharge (m^3^/sec) for each day of the year as measured at the USGS Milford Gauging Station on the lower Crawfish River (43°06’00" N, 88°50’58" W). Data plotted here are drawn from 1932 to 2020. The 2008 flooding corresponds to the sharp peak at days 168–170 (17–19 June). A secondary high peak, at about day 96 (5 April) records high spring flows in 2019. Note that the usual time of maximum discharge is between about day 67 and day 123 (2 March to 2 May). The day numbers of the maxima are approximate after day 59 due to the presence of leap years.

The hydrological record of the USGS Milford Gauging Station (USGS Station 5426000), located approximately 7 air km southeast and downriver of the Faville Prairie shows the magnitude and anomalous timing of the 2008 flood. A plot of the maximum average daily discharge (m^3^/sec) for each day of the year for the period 1931 to 2021 shows that the flood of 2008 not only produced the highest recorded daily discharge but also that the event occurred well into the 2008 growing season (Fig 2). This is a departure from the historical pattern of the higher flooding levels usually occurring in the late winter or early spring.

### Sampling for species occurrence and cover

This study is based on an array of 180 permanently marked points established by the late Dr. Max Partch as a part of his PhD research at the University of Wisconsin–Madison [26]. He resampled the points in 1978–79, (hereafter, 1978) providing the data that we compare to our post-flood resample. His points were placed in an evenly spaced rectangular grid with 100 ft (30.48 m) spacing between points. The lines are oriented to compass directions. The north to south dimension was assigned letters A (furthest north) to L. The west to east dimension was assigned the numbers 1 (furthest west) to 15 (Fig 1). Partch centered 2 x 2m^2^ plots on the points, an arrangement we duplicated. The 180 plots thus sample a total area of 720 m^2^, or 0.2% of the area of the unplowed portion of the site; and the distribution of plots a rectangle of approximately 429 x 337 m or 14.5 ha covering about 58% of the remnant. As noted above, the eastern edge of the site is formed by the Crawfish River, and as Partch’s interest focused on the prairie portion of the site, his array stopped approximately 200 m short of the river, excluding the most frequently flooded area.

Our resample occurred in two phases, 2010–2011 and 2015 (hereafter 2010). The second phase extended the sample into the wetter more frequently flooded portion of the remnant that is heavily invaded by native shrubs and trees and by the invasive strain of *Phalaris arundinacea* (reed-canary grass). In our resample, we divided the 2 x 2 m^2^ plot into four 1 m^2^ units (quadrats). The four 1 m^2^ quadrats were sampled in a clockwise direction starting with the southwest quadrat. Only species newly encountered were recorded in the three remaining quadrats. Here we report only the data for the entire 2 x 2m^2^ areas at each sample point.

We measured cover at each vegetation sampling point by a variation of the point intercept method. The unit being sampled was the two-dimensional visualization in the horizontal plane of the area encompassed by the crown of a species defined by the “polygon of minimal perimeter” also known in GIS usage as the “convex hull” of a set of points. The crown of an individual plant thus defined is visualized by connecting the outermost parts of the living foliage or branches. To record the “hits” a steel rod held vertically was lowered through the vegetation. A hit was noted if the rod intercepted the virtual canopy. This method does not require that a part of the plant be touched by the point. Sample points typically encountered more than one species. The hits were recorded in order from first encountered to last encountered. Only one hit per species was recorded even if more than a single virtual canopy was intercepted. Cover points were taken at the center of each of the four 1 m^2^ quadrats that comprised the occurrence sampling point (plot) resulting in a total of 720 cover points. If no plant canopies were intersected, the point was recorded as “bare” only one of which was encountered.

We used the revised USDA wetland indicator status (MWI, [27]) as a measure of the flooding tolerance of species. We used a numerical translation of the ratings with 5 assigned to the USDA “Obligate” (for occurrence in wetlands) rating and the other four ratings assigned numbers in descending order to “Upland”, assigned the value of 1. Species that did not have a value assigned were excluded from the analysis, but all species with a ranking in each quadrat were included. The mean number of species included in the comparison across both data sets was 17.0 (SD 7.61).

To evaluate the change in “conservation value” we used the “coefficient of conservatism” (COC) numerical rating as a measure of the conservation value of each species. These are the “C” values in the sense of Swink and Wilhelm [28]. Values range from 0 to 10, where a zero value indicates a species that is either non-native or associated with habitats that are degraded by human disturbances, and the higher values with species that are indicators of native habitats little affected by human disturbance [29]. Following [29] we used the mean COC value in our analysis. Our decision to use this metric is supported by a recent review that concluded that the CC provides a valid metric for evaluating the status of vegetation [30]

We collected species not identified in the field for later identification at the University of Wisconsin–Madison Herbarium. Our nomenclature follows that of the University of Wisconsin–Madison Herbarium, which is based on the latest revisions of North American Flora. For the analysis of the total taxonomic richness of the 180 sample plots we specify “taxa” because the count includes lumped categories for difficult genera. For example, although Partch recorded three species of *Carex* in his 1978 survey, most occurrences (88 out of 123) were recorded only as *Carex* sp. In our survey (2010) we identified 121 individuals to 11 species out of a total of 231 instances of *Carex* sp. presence. Similar discrepancies in field identification occurred with other genera, obliging us to use lumped categories. The data for both studies, with corrected and reconciled taxonomy were analyzed using JMP Version 15 (SAS Institute Inc., Cary, NC.). MATLAB (MathWorks, Nantick, Massachusetts) programs were created by the authors for data cleanup and specialized analysis.

The field work in this project was conducted under a research permit from the University of Wisconsin–Madison Arboretum (UW Arboretum), managers of the property officially owned by the Regents of the University of Wisconsin. The second author is an employee of the UW-Arboretum authorized to access all UW Arboretum properties.

## Results

### Total sample taxonomic richness

A total of 209 taxa, corrected for obvious errors as explained above, were recorded in the two surveys (Table 1). Of these, 89 (43%) were unique to one sample or the other. The 89 “missing” species were on average relatively uncommon (mean occurrence = 7.2 (SD 12.4) or 4% out of 180. The data for species missing in 2010 are, however, strongly affected by the complete absence of four formerly abundant prairie species; *Liatris pycnostachya* (prairie blazing star), *Sporobolus heterolepis* (prairie dropseed), *Primula meadia* (shooting star), and *Prenanthes racemosa* (glaucous white lettuce). These 2010 missing species had in the 1978 data occurrences of 84, 60, 37, and 32, respectively. Removing these reduces the mean occurrence of all the other missing species to 5.0 or 2.8% of 180.

**Table 1:** Number of species for each survey. Total taxon richness for both samples combined, species present in both, and unique to either the 1978 or 2010 sample. These numbers cannot be precise to the nearest species because of lumped categories and taxonomic difficulties.

Category recorded	Number of taxa
Combined for both surveys	209
Total the 1978 survey	148
Total in 2010 survey	181
Present in both surveys	120
Unique to the 1978 survey	28
Unique to the 2010 survey	61

Other species missing in 2010 are opportunistic weedy species some introduced (e.g., *Amaranthus retroflexus* [redroot Amaranth]. Of probable significance are potentially invasive species that occurred in 2010 but not 1978, which will be discussed below.

The 61 species present in 2010 but missing in 1978 have a mean occurrence per point of 4.86 (6.37 SD) and a range of 1 to 33 occurrences. Nineteen species had only one occurrence. The most common species, *Vitis riparia* (river bank grape), is a native wild grape with weedy tendencies that is widely dispersed. Most of its occurrences were of seedlings. Overall, the impact of the species newly added is not profound, with the possible exception of two invasive shrub species discussed below. Eight of the species are trees most of which were only present as seedlings. One of them, *Morus alba* (white mulberry), is a common nonnative invasive species not thought to be a major threat to prairies or marshes.

Although fewer species were recorded in the 1978 survey (148) than in 2010 (181) the ratio of native to non-native (introduced) species in the two samples was similar, 89% versus 92% for the 1978 and 2010 surveys respectively (chi-square = 0.93, 1 d.f., p = 0.34).

### Point results–Species richness, conservation status, and wetland indicator status

**Fig 3:**
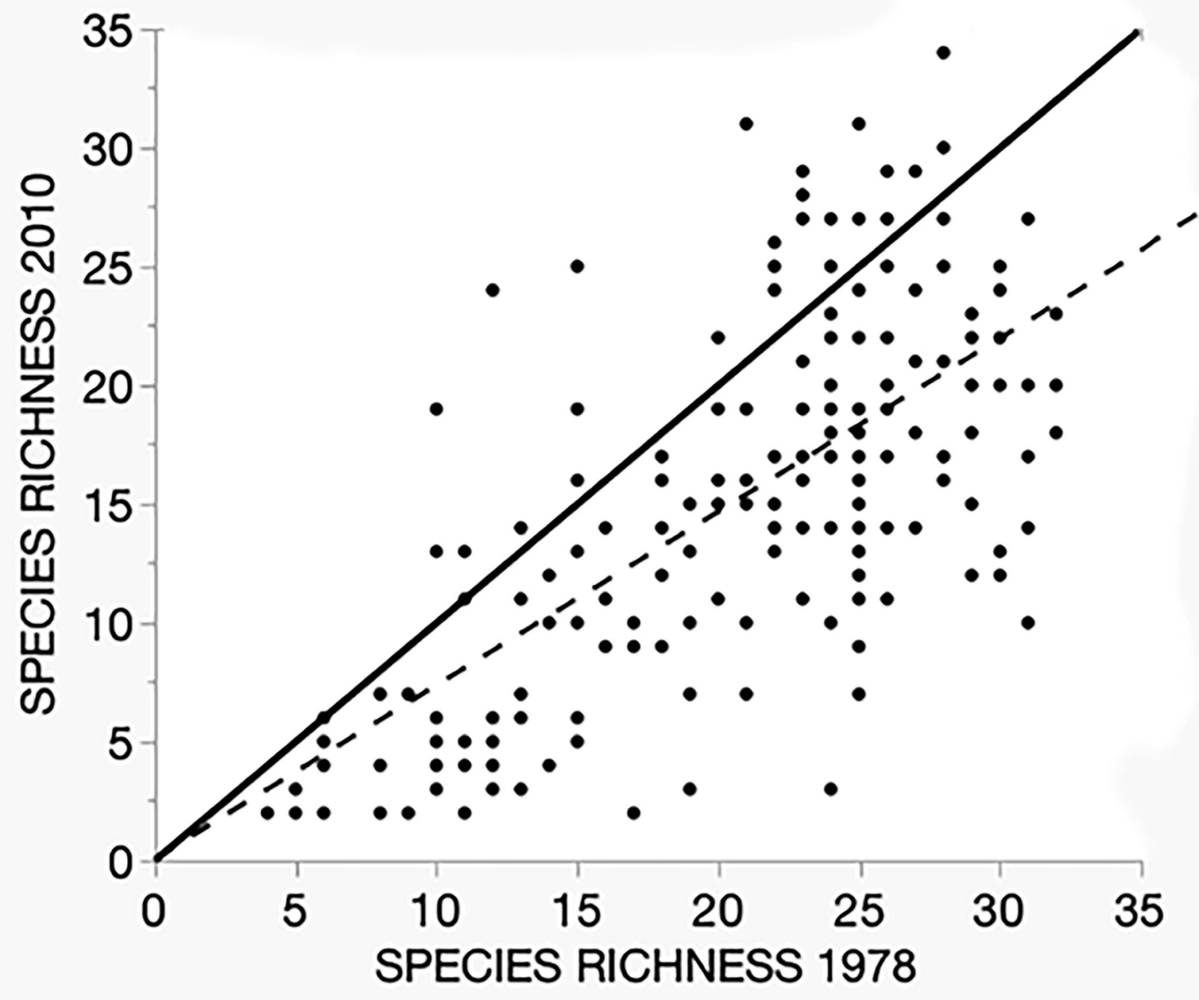
Species richness for each of the 180 sample points for the 1978 (X axis) and 2010 (Y axis) samples. Though highly variable, the overall relation is positive (dashed line, Y = 0.08 + 0.73X, R^2^ = 0.43, p < 0.0001, N = 180), indicating that a correlation between times was preserved despite an overall loss of richness. The slope of the regression reflects the greater absolute losses in the higher richness points.

We assessed change in point species richness by plotting values for each of the 180 points along two axes, the X axis being the richness observed in 1978 and the Y axis richness in the 2010 survey (Fig 3). A regression (dashed line) affirms that there is a correlation between richness at the two surveys, with an R^2^ value of 0.43 (p <0.0001). However, the visual evidence of the distribution of points relative to the “line of no difference” (LND, solid line) which separates points that lost richness (below the LND) from those that gained richness (above the LND) is more informative. Clearly far more points lie below it than above, 149 to 31, with an overall mean loss per point of -5.5 (SD 6.2). The paired difference in point richness between the surveys, 20.7 (SE 0.53) for 1978 and 15.2 (SE 0.59) for 2010 (paired t = - 11.8, 179 d.f, p < 0.0001) provides statistical confirmation of the difference. We attribute the greater part of this pattern to the consequences of the extreme flooding.

**Fig 4:**
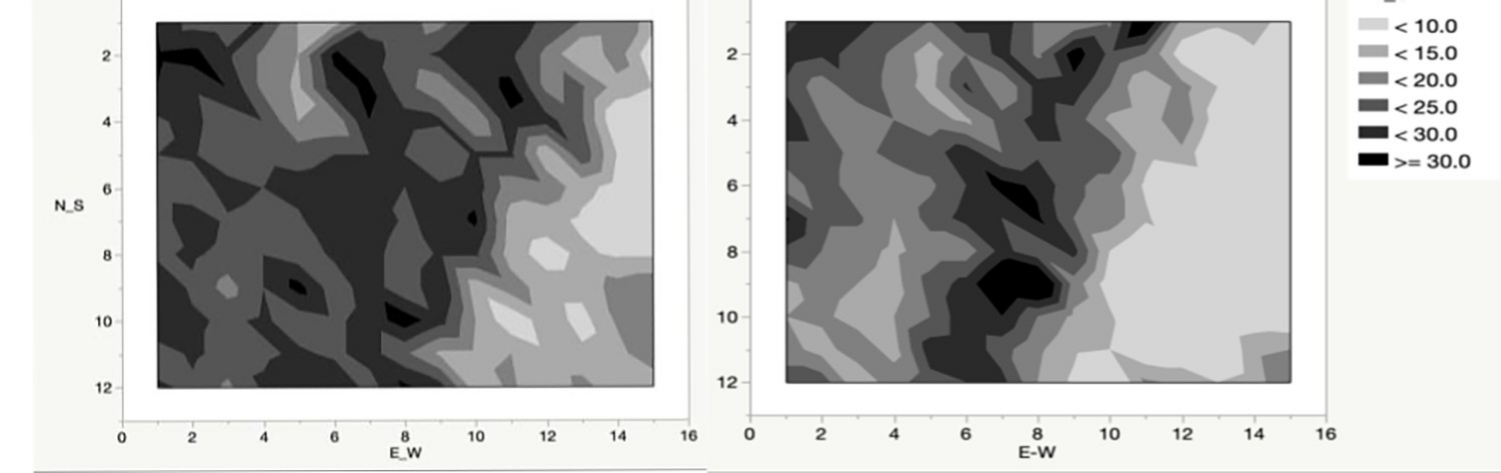
Contour plots of point species richness for the 1978 survey (left panel) and 2010 survey (right panel) for the entire 180 plot array. The plots are arranged with north at the top. Note the strong expression of the ridge in the data for both surveys. The 1978–2010 comparison also shows the expansion of the low diversity portions of the site, especially in the easternmost portions of the site, reflecting the general loss of alpha-level richness over the entire site. Note that the eastern limit of the point array is about 200 m short of the summer position of the Crawfish River.

Contour plots of the spatial distribution of richness in which the darker tones indicate greater species richness show spatial differences between surveys. In both surveys a region of higher richness can be seen running diagonally across the site (Fig 4). This corresponds to the presence of the ridge that crosses the site angled approximately 17 degrees east of north. This modest elevational difference provides drier habitat important for some of the prairie specialists. The zone of higher richness west of this ridge, which is very apparent in the 1978 data, is much diminished in 2010. The area of lowest richness in the easternmost portion of the site can be seen in both surveys but is expanded in the 2010 survey. While a part of this may be due to the flooding, it is equally likely that the expansion of woody vegetation that has occurred over decades in this part of the site is responsible for much of the change.

**Fig 5:**
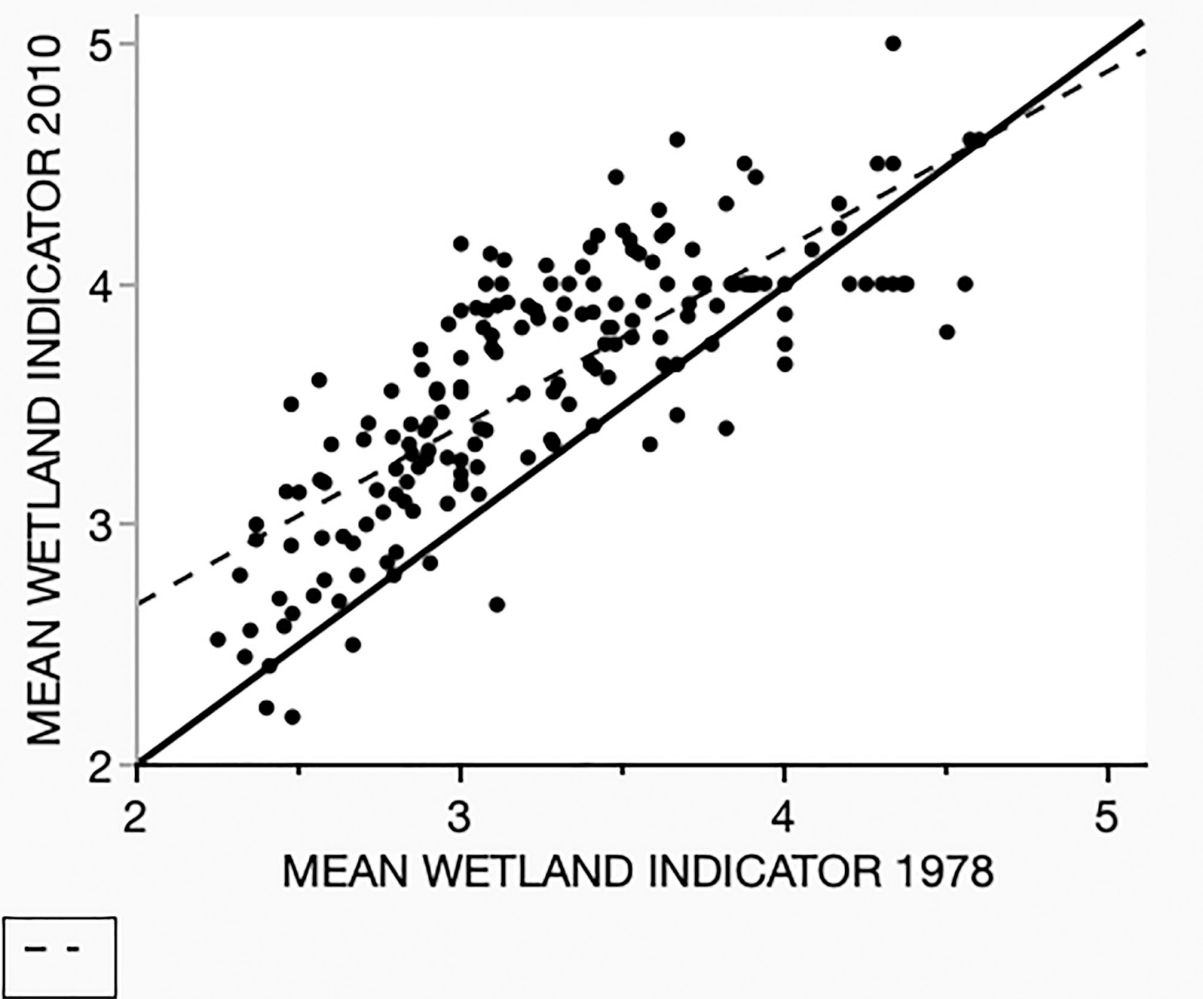
The mean wetland indicator value (MWI, see methods), the 2010 survey (Y) against the MWI for the 1978 survey (X), by point (N = 180). Species were assigned an integer value from 5 (Obligate wetland species) to 1 (Obligate upland species). The dashed line is a regression (Y = 1.19 + 0.74X, R ^2^ = 0.63, p <0.0001.). The solid line is the line of no difference (i.e., Y = X). Most of the points lie above this line, indicating that they had increased mean values in 2010, that is, greater representation of wetland tolerant species.

A flood is an extreme in the variation of site moisture conditions. An expectation following from this is that there would be a relative increase in the occurrence of wetland tolerant species. This is borne out by the change in the mean wetland indicator (MWI) status of each point (Fig 5). As with richness, the resulting scatter diagram shows a relationship between the two samples, with an R^2^ of 0.60 (p < 0.001) indicating the preservation of much of the site-wide correlation of wetland tolerance species. The important pattern is the distribution around the LND which shows a significant shift in MWI. The mean MWI increased by 0.33 (SE 0.03) with higher values indicating greater wetland tolerance. (Paired t = 12.5, d.f. = 179, p < 0.0001). The fact that the regression has a slope less than 1 (0.68) and that it intersects the LND at the highest MWI values shows that the shift toward a greater relative occurrence of wetland species is more pronounced for points that had the lowest MWI values in the 1978 survey.

**Fig 6:**
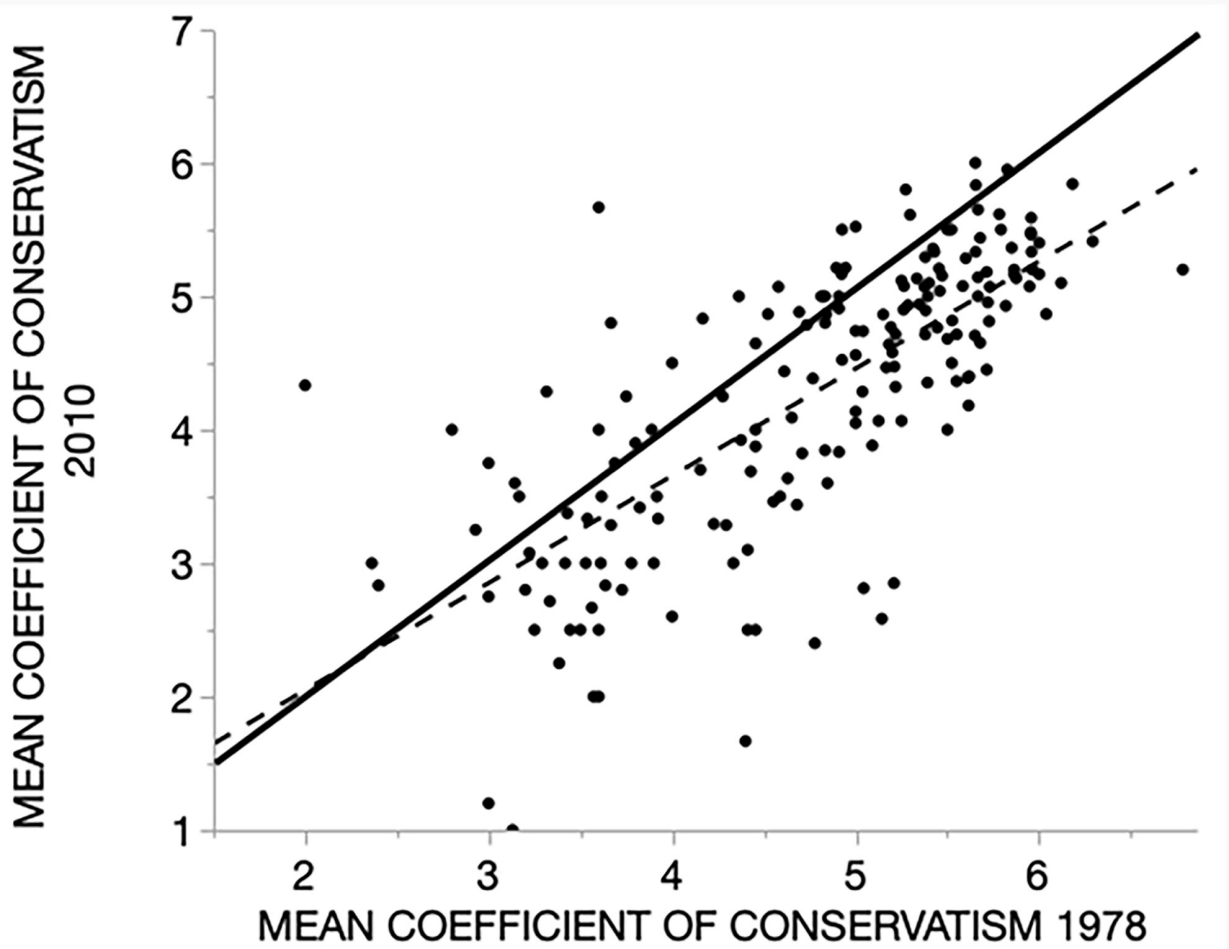
Comparison of the mean values of the “coefficient of conservatism” for each point in the two surveys (N = 180). Higher values indicate more value for biodiversity conservation. The dashed line is the regression (Y = 0.44 + 0.80X, R^2^ 0.53, p < 0.0001) and the solid line a reference line of no difference).

To determine if the changes indicated in Figs 4 and 5 are meaningful for conservation, we compared the mean coefficient of conservatism value (COC) of each point for the two surveys. This relation, like that of species richness, is positive and significant (dashed line, Fig 6) confirming that the underlying controls of conservation value are to some degree preserved. As with richness, most of the lines are on the loss side of the LND (solid line, Fig 6). Accordingly, the mean point values of COC declined, 4.8 ± 0.07 for the 1978 survey and 4.3 ± 0.08 for 2010 survey. The difference in these values of -0.49 represents a significant drop on the 0–10 scale of the COC (paired t = 9.0. d.f. = 179, p < 0.0001). Both the species richness and the conservation value of the site have declined.

### Species results

**Fig 7:**
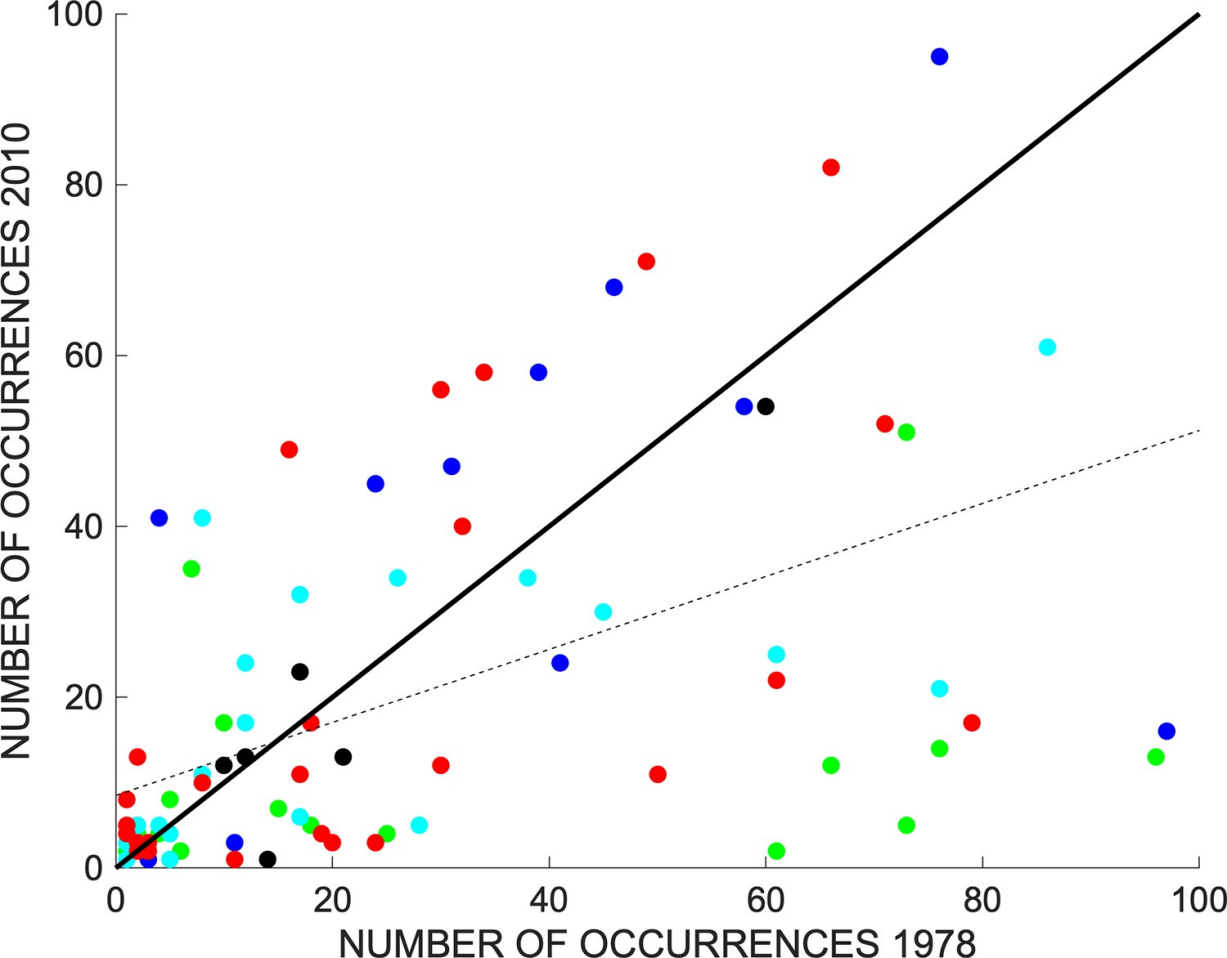
The occurrences of the 89 species that were present in both samples. Lumped taxa (e.g., *Carex* spp.) are excluded. The dashed line is the linear regression on the untransformed data, and the solid line the line of no difference. Although the variance around the regression line is large, the overall the trend is positive and significant (Y = 8.0 + 0.45X, R^2^ = 0.30, p < 0.0001). The colors indicate the Wetland Indicator Status; Red = Obligate wetland, Green = Facultative wetland, Cyan = Facultative, Dark Blue = Facultative upland, Black = Upland.

There is a wide range of change in the absolute number of occurrences of the individual species (Fig 7). Statistically, however, species occurrence in the two data sets are significantly related. Although a liner regression is nominally significant (R^2^ = 0.28, p < 0.0001) applying a log transformation provides more appropriate evidence of a trend with R^2^ = 0.42, lnY = 0.81 + 0.59 lnX, p < 0.0001. The slope of the regression is less than one, an indication that the vegetation has been reorganized as well as diminished. However, it is the distribution of data points around the LND with more below the line than above it that most clearly reveals the change.

Further statistical confirmation of the reduction in occurrences is provided by a paired t-test evaluating the hypothesis that the average mean difference in species occurrence for log transformed data is zero. This hypothesis is rejected, with a mean difference in ln(occurrence) of -0.38 (t = -3.39, p > |t| = 0.001 and a correlation of +0.65.

We considered the 22 species that were removed for the paired analysis because they were absent in one of the two surveys. These are almost evenly split between “added species” and “lost species.” The average number of occurrences for species in this group was 25.9 ± 6.9 for the 1978 survey and 20.3 ± 4.9 for 2010, the lower number being in accordance with the general trend for lower occurrences in the 2010 survey. Four of the 12 lost species from the 1978 survey are nonnatives, and none are woody. Another four losses were for characteristic native prairie species and were discussed above.

The 2010 survey added 10 species of which five were woody species, two of which are the invasive non-native species *Rhamnus cathartica* (common buckthorn) and *Frangula alnus* (alder buckthorn). Both species are widely distributed over the site but were at the time of sampling mostly seedlings and small saplings 5 to 10 years or more short of reproductive maturity. A native species in this group, *Salix petiolaris* (meadow willow), is a shrubby species that can grow to 2 m or more in height. Its increase does preserve native quality, but at a loss of alpha-level species richness owing to its shading effect. On wetter parts of the site it, along with other woody species, can form an almost continuous canopy suppressing herbaceous species below it.

Using the complete data set (N = 209), we lumped the species into “increased” and “decreased” categories (Table 2). The absolute value of the mean of the decreasers is greater than the mean gain of the increasers. The losers lost more than the increasers gained. These data are, of course, another way of looking at the overall pattern of a loss of species richness at the point level.

**Table 2:** Occurrence of species that increased and decreased across surveys.

Type of Change	Number of Taxa	Mean Occurrence 1978	Mean Occurrence 2010
Increased	92	9.4 ± 2.0	17.8 ± 3.0
Decreased	117	29.2 ± 3.2	9.7 ± 1.5

The mean occurrence out of 180 points for the group of species that showed positive change (Increased) or negative change (Decreased) in number of occurrences between the two surveys. Values are means plus or minus the standard error.

**Fig 8:**
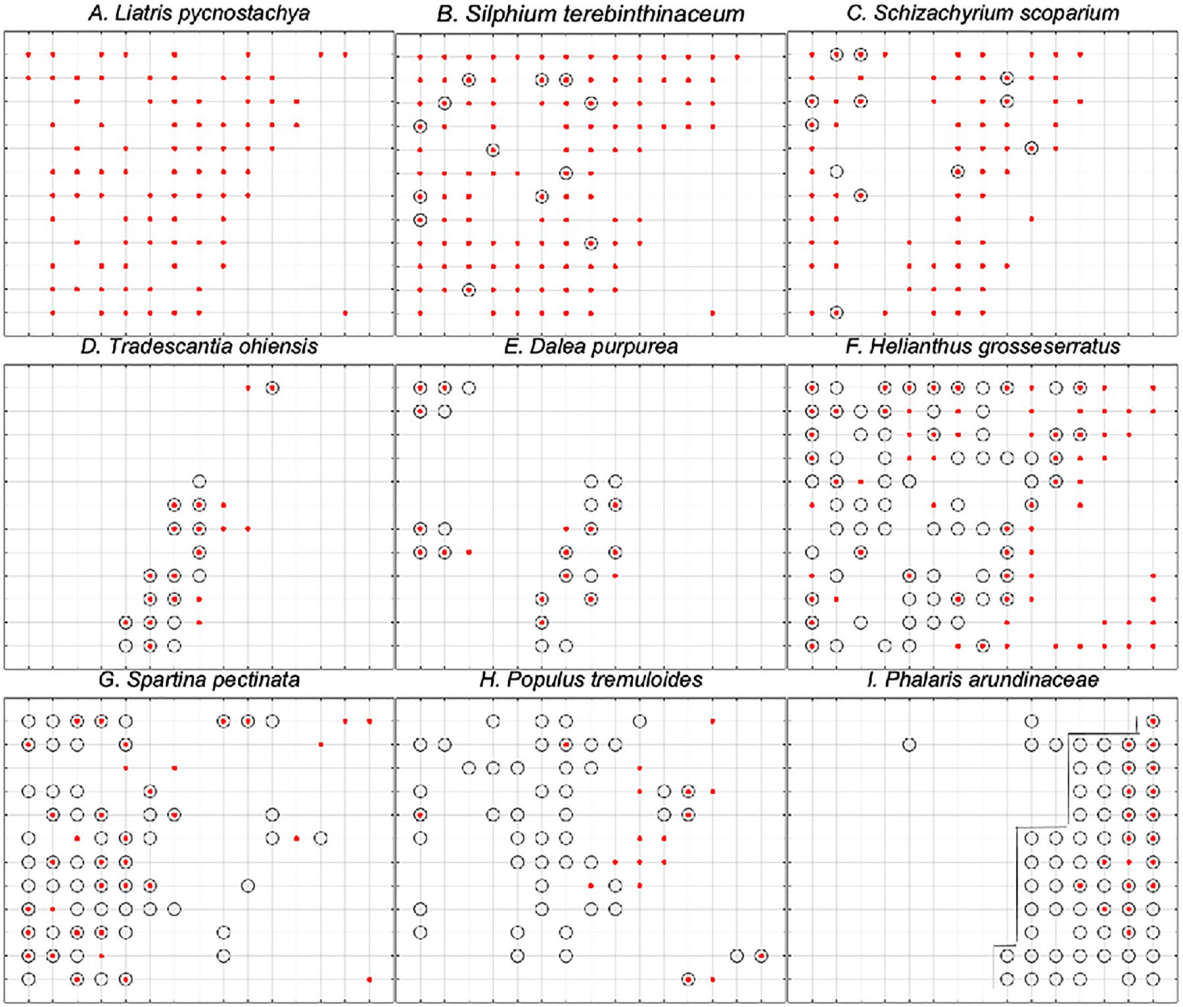
Maps of individual species showing their distribution within the 180 points in 78–79 (small red points) and in 10–15 (open circles). Note the high site fidelity shown in panels D and E. The lines in the *P*. *arundinacea* graph (panel I) indicate the split used to define the regularly flood affected portion of the sampling array (see text).

Maps of individual species distributions in the two samples show the spatial patterns of increase or decrease (Fig 8). The map for *L*. *pycnostachya* (Fig 8, panel A.) illustrates the complete loss of this formerly abundant species within the sampling array. As discussed above, similar patterns could be shown for *S*. *heterolepis*, *P*. *media*, and *P*. *racemosa*. We now know that of these four species only *P*. *racemosa* has so far not been observed on the site in or outside of the sample points. The other three are present in very low abundance.

*Silphium terebinthinaceum* (prairie dock, panel B) is an example of catastrophic decrease short of extinction within the permanent points. Its number of occurrences dropped from 108 in 1978 to 14 in 2010 with all of these being at points where it was recorded in the earlier survey. A recent partial resurvey has shown that this species is recovering and has reoccupied some of the points from which it was absent in the second survey as well as occurring at a few new points (Kennedy 2020).

*Schizachyrium scoparium* (little bluestem, Panel C), a mid-statured prairie grass, lost 50 of its occurrences, surviving at only 12 points. All but one of these were at points formerly occupied.

Some prairie species were little affected in either their spatial distribution or abundance. *Tradescantia ohiensis* (Ohio spiderwort, Panel D), and *Dalea purpurea* (purple prairie clover, Panel E), both characteristic prairie species, show notable persistence. The distribution of both species correlates with the ridge portion of the site (Fig 3). *Viola pedata* (birdfoot violet, not plotted) is another example of a prairie species that increased slightly from 10 to 12 occurrences and substantially retained its spatial distribution. The persistence of these species shows that some prairie species have an unexpectedly high capacity to survive flooding. None of these species is of high stature, and so would have been among the last species to rise above the flood level, if they had had any aerial portions surviving the flood.

*Helianthus grosseserr*a*tus* (sawtooth sunflower, Panel F) is a native species that is common along roadsides and other modified habitats; a fact recognized by its low COC value of 2. Its pattern of habitat loss suggests an interaction between the acute growing season flood of 2008 and the usually spring-flooded easternmost portion of the site. To explore this, we broke the data into a “wetter” and “drier” portion based on the occurrence of *P*. *arundinacea* a notoriously aggressive invader of wetlands [31] (Fig 8, Panel I). This divides the data into a block of 60 wetter (W) quadrats subject to the repeated flooding of the Crawfish River, and 120 drier (D) quadrats.

ANOVA using the natural log transformation of species richness as the dependent variable and drier-wetter (D, W) and their interaction as the independent variables shows that the species richness differs between these two groups. All the factors including the intercept were significant (all with p < 0.0001, overall F = 180.3, d.f. = 3,356). Comparison of the mean ln(richness) values using Tukey’s HSD, showed all four to be significant with p < 0.0001. The results for an untransformed ANOVA on species richness, despite a non-significant interaction, produced similar results in the post hoc analysis.

### Cover measured in the 2010 survey

The cover data show that the site was densely vegetated in 2010 despite the effects of the flood. We recorded only one “bare” cover point out of the 720 cover points taken (Table 3). The remaining 719 points encountered predominately herbaceous species with woody species (six tree species and 14 species or species groups of shrubs) accounting for 19.3% of the cover (Table 3). Within the herbaceous group, grasses and *Carex* sp. accounted for 52.2% of the cover, with *Carex* sp. at 19.6% and grass species at 32.5%. The fact that the invasive *P*. *arundinacea* was the second most encountered taxon, at 25.6% cover shows the degree to which this species has commandeered space (Table 3). Removing the dubiously native species *P*. *arundinacea* and *Phragmites australis* (common reed) reduces the native dominance to 88.7%.

**Table 3:** Percent cover for the 2010 survey.

Species/Taxon	% Cover
*Carex sp*.	37.9
*Phalaris arundinacea*	25.6
*Eleocharis compressa*	17.2
*Calamagrostis canadensis*	12.6
*Salix petiolaris*	11.2
*Andropogon gerardii*	10.6
Grass sp.	8.3
*Spartina pectinata*	8.3
*Spirea alba*	6.9
*Carex stricta*	6.5
*Helianthus grosseserratus*	6.1
*Salix spp*.	5.3
*Anemone canadensis*	5.1
*Lysimachia quadriflora*	4.7
*Populus tremuloides*	3.7
Remaining 79 species	26.1

Percent cover of the 15 most encountered species or species groups. There were four cover points at each 4 m^2^ point for a total of 720 cover samples. There were 1659 species or species group “hits” and one hit for “bare ground”. The sum of the percent occurrences of all species is greater than 100 due to the overlap in plant canopies (see Methods).

### Non-native and invasive species

Two general compositional changes that have occurred since the 1978 Partch survey are the advance of woody vegetation and the expansion of invasive nonnative species. Two species figure prominently in these changes–the native *Populus tremuloides* (quaking aspen) and the wetland invasive *P*. *arundinacea*. Native willows of at least five species (*Salix* spp.) are not invaders because they have long been present on or near the site. They have, however, increased from 94 to 120 occurrences between surveys.

*P*. *arundinacea* is considered a menace to biodiversity [31–33]. It was recorded at 20 points in the 1978 survey and at 62 points in 2010. There was a spatial overlap of 19 points between the two surveys. The pattern of change in species richness at points containing *P*. *arundinacea* confirms these observations. The species richness in *P*. *arundinacea* plots in the 2010 was lower in the 19 overlap points but higher in the 43 new points, 4.9 ± 1.10 versus 9.2 ± 0.75, a value that is well below that of the average 2010 richness for points lacking *P*. *arundinacea*.

*P*. *tremuloides*, a native tree species (Fig 8, Panel G), also poses a threat to the biodiversity of the Faville Prairie. Its occurrence, despite attempts to limit its spread [34], has more than doubled from 18 to 48 plots over approximately 36 years. Most of these new occurrences are less than 2–3 m tall, prevented from maturing to tree size only by frequent burning and intermittent mechanical and chemical control.

## Discussion

We have shown that the vegetation of the Faville Prairie has changed dramatically since the 1978 survey. The changes we have documented include both those that have accumulated leading up to the 2008 flood and the effects that followed from it. We have confirmation that in the western portion of site the changes up to 2005 were small. They did not include the extinction of any of the abundant prairie species within the sample array and in general the authors found relatively little change “The majority of species density increases were attained by the 1976–1978 sampling period and remained high since” [14, 24, 35].

The story is different in the eastern margin adjacent to the Crawfish River. When Partch established the array in the 1940s he ended it about 200 m short of the river’s edge, presumably because beyond this point the vegetation shifted to marsh. Aerial photos show that there was very little tree cover at that time. In ensuing years, the tree and shrub cover has expanded, causing species with prairie affinities to have largely disappeared. A result of the increase in woody cover is illustrated for *H*. *grosseserratus* (Fig 8F, lower right). Its loss from this area cannot reasonably be attributed to the 2008 flood because of its robust recovery in most of the prairie, being recorded at 62 of the 180 points. The changes of most concern, the pattern of the reduction in occurrence and complete extinction of some prairie species recorded in the drier portions of the site, are most reasonably explained by effects of the extreme flood.

Despite the negative effects of the flood, there is evidence of resilience, by which we mean the ability of an ecosystem to recover after disturbance to a state approximately the same as its pre-disturbance condition. The negative effects were most pronounced because of the losses to valued native species (e.g., *L*. *pycnostachya*) and the failure to reduce the expansion of undesirable invasives. Significantly, however, to date only one species of high conservation value, *P*. *racemosa* (COC = 9) appears to be extinct on the site. The 2010 cover data show that there was robust re-occupancy of the site and thus retention of photosynthetic function, albeit at reduced species richness. The resilience of some prairie species (e.g., *T*. *ohiensis*, Fig 8D) was shown by their ability to survive in place. A recent study revealed that two species of *Silphium* that had significant flood-related loss have expanding populations [36]. Thus, as an assemblage, the plant community of the Faville Prairie remnant appears able to hold the site by virtue of its mixture of more and less wetland tolerant species. It has the capacity to survive a single extreme flood, and so, in Leopold’s terms, to “keep (almost) all the parts” [37].

It is the morphological and physiological traits of the species that determine the community response. The concept of “hydrological niche” [38] is relevant here, both to pre-flood distribution and the flood response. The change in the distribution of species richness illustrates the operation of microhabitat selection and the disturbance to this pattern caused by the flooding (Fig 3). The differential survival of species along the flood tolerance gradient disrupted, but did not totally obscure, the micro-topographic patterns.

We lack detailed information on the flood tolerance of most species but can cite observations that point to some of the ways the Faville species survived flooding: 1) Survival by having photosynthetic tissues above the flood waters. 2) The capacity for intact plants to survive total inundation, perhaps because of an ability to switch their metabolism towards that of an aquatic plant. 3) The ability of underground regenerative structures to endure low oxygen conditions and resprout after the flood recedes. 4) A seedbank that will survive inundation and permit recovery in post-flood conditions.

If a species can maintain functioning leaves and stems above the flood water, its prospects for survival are significantly improved [21]. This was evident for many of the woody species that presumably possessed the adaptations general to woody plants of flooded environments [39, 40]. Surviving species included shrubby and tree willows (*Salix* spp.), *Cornus spp*. (dogwood), and three tree species (*Quercus velutina* (black oak), *Fraxinus pensylvanica* (green ash); and *P*. *tremuloides*). Shrub species with canopies in the 2 to 3 m maximum height range (e.g., *Cornus* spp.) retained their leaves above the maximum flood level, but lost them where inundated, creating a sharp line post flood that marked the level of high water. *P*. *tremuloides* of tree size suffered some mortality, but the recovery of individuals less than 2 m in height was robust, indicating a degree of flood resilience documented for related species [41].

A few species were able to survive complete inundation intact, emerging from the flood seemingly little affected. *Maianthemum stellatum* (starry false Solomon’s seal) for example, was conspicuous across large areas immediately post flood as documented in a photo taken on 7 July 2008. Given its classification as a “hydrophyte” and a “facultative wetland” species [42] this level of survival is expected. The species also had a remarkable spatial persistence. Its occurrences increased between surveys from 12 to 19. Its ability to persist is supported by the fact that 11 of the 19 2010 occurrences were in the same plots as the 1978 survey, a spatial recurrence rate of 92%.

*Andropogon gerardii* (big bluestem) is a widely distributed species of broad tolerance, and this seems to extend to its ability to survive flooding. We recorded 63 occurrences (42% frequency) which was, however, a 29% reduction from the 1978 data. In contrast, data collected on the Faville Prairie in 50 quadrats in 2007 and again in 2009 indicate an increase in frequency from 24% to 58% over that two year period [43]. We have no explanation for the demographic processes (e.g., establishment from seed, clonal expansion) responsible for this degree of expansion. We do, however, have immediate post-flood information (a photograph) that *A*. *gerardii* is a species whose above-ground portions can survive flooding. Thus qualitatively, it is a species unlikely to be driven to local extinction even by frequent floods.

*Eryngium yuccifolium* (rattlesnake master), a species present at Faville but not found in the permanent plots, also survived inundation. A bloom of whitish calcium deposits was observed on its surviving leaves, an indication of underwater photosynthesis [44, 45]. The flooding tolerance of *E*. *yuccifolium* is expected given the wetland affinities of many other species in the genus [46, 47].

Many species that survived flooding lost their above ground leaves and stems and recovered from below ground structures. For example, though only dead leaves of *Heuchera richardsoniana* (prairie alumroot) were seen immediately after the flood, mature plants were observed in our post flood resample. Its recovery, however, was only partial. Its distribution on the site in 1978 is very similar to that of *M*. *stellatum*, but unlike that species its abundance was reduced from 31 occurrences to only five in the 2010 survey, four of which were at points where it had been recorded earlier. *D*. *purpurea* is another example of a species that was absent above ground after the flood bur recruited from the seedbank and likely also from below ground structures [48].

The survival of *Platanthera leucophaea (*eastern white fringed prairie orchid), a species known to be present on the Faville Prairie but not recorded in either survey, deserves special mention. Its status as “endangered” in Wisconsin, and as “threatened” Federally accounts for the concerns for its survival [49]. Though we have no data on this species (except zeros), unpublished information from the Madison Audubon Society corroborates our observation. No individuals were present on the site in 2008 or 2009. It did, however, reappear outside of our plots in 2010. The absence of above ground stems for two years was not surprising, since this species, like many other orchids, exhibits “prolonged dormancy” the ability to remain dormant for one or more growing seasons. Though the advantages of this behavior are still not entirely clear, the onset of dormancy appears to be prompted by stress [50]. It may reasonably be surmised that the stress of flooding was the cause of its post-flooding absence [50]. The prolonged dormancy trait may therefore be another means by which species can be resilient in the face of extreme floods.

The graminoids as a group, defined here as either Poaceae or Cyperaceae, responded as one might expect, with the Cyperaceae showing evidence of superior flood tolerance [51, 52]. Poaceae as a group declined from 573 total occurrences to 424, whereas the Cyperaceae increased slightly from 213 to 220, changes of -26% and +3%, respectively. The survival of grass species varied from complete loss for *S*. *heterolepis*, to an increase of 71% in occurrences for *Spartina pectinata* (prairie cord grass; Fig 8G).

Rooney and Leach comment on the substantial changes in C3 graminoids and C4 grasses over the 1948–2004 interval [14]. In our data the pattern was mixed. Looking only at the grass-sedge (*Carex*) component, we found increasers and decreasers in both categories. The averages of five species in both the C4 and C3 categories, however, indicated an average increase of 19.0 occurrences for the C3 species (grasses plus sedges) versus an average drop of -22.4 occurrences in the all-grass C4 species. These numbers are too small for statistical analysis, but the difference suggests that C4 species may be more susceptible to flooding stress than C3 species.

### Long-term prospects

**Fig 9:**
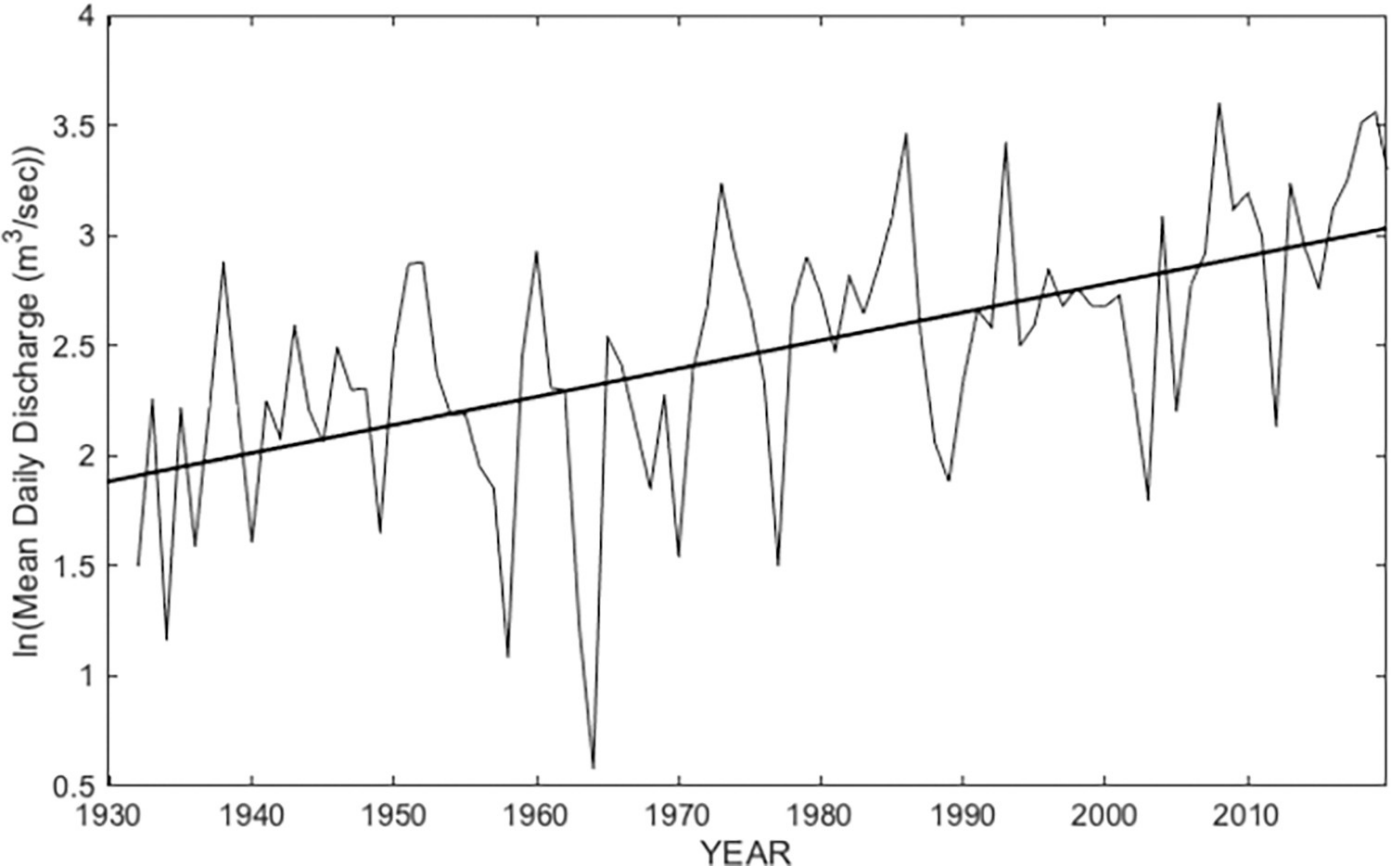
Natural logarithmic transformation of mean annual discharge (m^3^/sec) of the Crawfish River as recorded at the USGS Milford Gauging Station (1931–2019). The data and the regression line have little value for predicting discharge for any single year, but a strong trend (R^2^ = 0.34, p < 0.0001, N = 88). These data do not reflect seasonality of flows, which is important for determining flooding effects on the vegetation (Fig 2). The data are too few to conclude that growing season floods are increasing in frequency.

It seems likely that the Faville Prairie will be able to recover towards its preflood condition, provided that growing season floods of the magnitude of the 2008 flood will be as rare as the historical data (Fig 2) suggest and no other catastrophic disturbances occur. If, however, extreme growing season floods should recur separated by one or a few years, they could cumulatively move the system towards a less diverse wetland, likely one heavily invaded by invasive species [20]. There are reasons for concern. Evidence is growing that climate change continues, and there are indications that rainfall regimes may be shifting. Studies in other regions and globally have concluded that extreme rainfall episodes will become more frequent [18, 53–55]. There are also regional indications of climate changes. The precipitation record in Wisconsin since 1950 has shown an increase of 10–15%, with most of this occurring in the lower three quarters of the state [56]. Data on annual mean daily discharge of the Crawfish River from the USGS Milford station show an increasing trend, and two of the largest floods have occurred in the last 14 years (Fig 9)

There is, however, more than rainfall involved in extreme floods. Two of the factors that have been identified are especially relevant to the Crawfish River: a) antecedent soil moisture within the watershed, b) areal extend of the watershed [57]. A third factor, important for its effect on natural ecosystems is the season of the flood. Rainfall was, of course, the proximate cause. New record rainfalls for a 24 hour period were set for four stations in the southern tier of counties of Wisconsin ranging from 11.4 to 15.1 cm with over 150 km the maximum distance between the four stations [15]. Rainfall at these levels would cause flooding whatever the antecedent conditions, but in this instance the months leading up to the “supercell” event were preceded by snowfall of over 250 cm and a spring rainfall that together led to saturated soils [15] This maximized the amount of water flowing into the creeks and rivers. The relatively small area of the Crawfish watershed (1974 km^2^) meant that the zone of high rainfall covered all of the watershed. Finally, that the rainfall event occurred in late spring when the prairie vegetation was well advanced ensured that the flood killed all or most of the aboveground portions of a large proportion of the species. We can say that if the flood had occurred in late winter prior to the onset of growth it would have had a much less profound effect. We cannot say what effect a flood of 2008 levels would have had at various times after species had broken dormancy, but an even later flood might be more destructive.

For a flood comparable to that of 2008 to occur a similar confluence of prior conditions and extreme events must occur. We have no way of calculating how likely this will be and only rough guesses are possible. Given what is reasonably well established, we can conclude that the probability of another “2008 flood” is greater than zero. Estimating the distribution of times between floods is subject to even greater uncertainty. As a practical matter, then, the managers of the Faville Prairie are reduced to those measures that will ensure healthy populations of the most valued species, and that will have value with or without the threat of more extreme floods. These include pushing back the tree and shrub invasion that threaten the biodiversity of the site and maintaining a burning management program. Our results and the observations that have been made in the following years suggest that the wet prairie vegetation of the Faville Prairie has the capacity to absorb disturbances and recover to maintain, so far, all (or nearly all) of the plant species present when the reserve was created in the 1940s. The restoration of some of the missing animal species, such as greater prairie chickens (*Tympanuchus cupido*) will probably require the restoration of more land and greater connectivity. This may not be an unreachable dream. The Madison Audubon Society has ongoing prairie restorations on 352 ha of land near the Faville Prairie remnant. One parcel shares a boundary with the south side of the Faville Prairie. The presence of these parcels will be able to sustain a greater variety of prairie, oak savanna, and wetland ecosystems including insects and small mammals, and perhaps in the future, the iconic prairie chicken. The additional populations of native species, some of them on sites that lie above the 2008 flood, increases the likelihood that the historical Crawfish Prairie remnant and the surrounding restorations, will survive future climatic threats [58].
